# Update Systematic Review, Meta-Analysis and GRADE Assessment of the Evidence on Parastomal Hernia Prevention—A EHS, ESCP and EAES Collaborative Project

**DOI:** 10.3389/jaws.2023.11550

**Published:** 2023-08-29

**Authors:** Alexander A. Tzanis, Cesare Stabilini, Filip E. Muysoms, Lisa Rossi, Ourania Koutsiouroumpa, Dimitris Mavridis, Michel Adamina, Umberto Bracale, Henk-Thijs Brandsma, Stéphanie O. Breukink, Manuel López Cano, Samantha Cole, Suzanne Doré, Kristian Kiim Jensen, Marianne Krogsgaard, Neil J. Smart, Christoffer Odensten, Chantal Tielemans, Stavros A. Antoniou

**Affiliations:** ^1^ Metaxa Memorial Cancer Hospital, Piraeus, Greece; ^2^ Department of Surgery, University of Genoa, Genoa, Italy; ^3^ Department of Surgery, Maria Middelares Hospital, Ghent, Belgium; ^4^ Department of Surgery, IRCCS Policlinico San Martino, University of Genoa, Genoa, Italy; ^5^ Department of Primary Education, School of Education, University of Ioannina, Ioannina, Greece; ^6^ Department of Surgery, Cantonal Hospital Winterthur, Zurich, Switzerland; ^7^ Department of Public Health, University of Naples Federico II, Naples, Italy; ^8^ Department of Surgery, Antonius Ziekenhuis, Sneek, Netherlands; ^9^ Maastricht University Medical Centre, Maastricht, Netherlands; ^10^ Abdominal Wall Surgery Unit, Val d’ Hebrón University Hospital, Universidad Autónoma de Barcelona, Barcelona, Spain; ^11^ Patient Representative, Nottingham, United Kingdom; ^12^ Patient Representative, Rayne, United Kingdom; ^13^ Digestive Disease Center, Bispebjerg University Hospital, Copenhagen, Denmark; ^14^ Department of Surgery, Zealand University Hospital, Koege, Denmark; ^15^ Department of General Surgery, Royal Devon and Exeter Hospital, Exeter, United Kingdom; ^16^ Department of Surgical and Perioperative Sciences, Surgery, Umeå University Educational Unit at Sunderby Hospital, Sunderby, Sweden; ^17^ University Hospital Ghent, Ghent, Belgium; ^18^ Department of Surgery, Papageorgiou General Hospital, Thessaloniki, Greece

**Keywords:** stoma, ostomy, colostomy, mesh, prevention

## Abstract

**Objective:** To perform a systematic review and meta-analysis on the effectiveness of prophylactic mesh for the prevention of parastomal hernia in end colostomy, with the ultimate objective to summarize the evidence for an interdisciplinary, European rapid guideline.

**Methods:** We updated a previous systematic review with *de novo* evidence search of PubMed from inception up to June 2022. Primary outcome was quality of life (QoL). Secondary outcomes were clinical diagnosis of parastomal hernia, surgery for parastomal hernia, and 30 day or in-hospital complications Clavien-Dindo ≥3. We utilised the revised Cochrane Tool for randomised trials (RoB 2 tool) for risk of bias assessment in the included studies. Minimally important differences were set *a priori* through voting of the panel members. We appraised the evidence using GRADE and we developed GRADE evidence tables.

**Results:** We included 12 randomized trials. Meta-analysis suggested no difference in QoL between prophylactic mesh and no mesh for primary stoma construction (SMD = 0.03, 95% CI [−0.14 to 0.2], I^2^ = 0%, low certainty of evidence). With regard to parastomal hernia, the use of prophylactic synthetic mesh resulted in a significant risk reduction of the incidence of the event, according to data from all available randomized trials, irrespective of the follow-up period (OR = 0.33, 95% CI [0.18–0.62], I^2^ = 74%, moderate certainty of evidence). Sensitivity analyses according to follow-up period were in line with the primary analysis. Little to no difference in surgery for parastomal hernia was encountered after pooled analysis of 10 randomised trials (OR = 0.52, 95% CI [0.25–1.09], I^2^ = 14%). Finally, no significant difference was found in Clavien-Dindo grade 3 and 4 adverse events after surgery with or without the use of a prophylactic mesh (OR = 0.77, 95% CI [0.45–1.30], I^2^ = 0%, low certainty of evidence).

**Conclusion:** Prophylactic synthetic mesh placement at the time of permanent end colostomy construction is likely associated with a reduced risk for parastomal hernia and may confer similar risk of peri-operative major morbidity compared to no mesh placement. There may be no difference in quality of life and surgical repair of parastomal hernia with the use of either approach.

## Introduction

Parastomal hernia constitutes the most common complication following the construction of an end colostomy, occurring in up to 50% of cases in the long-term [1]. Surgical repair is warranted in patients who experience acute parastomal hernia complications or those with chronic symptoms that impair quality of life.

Prophylactic reinforcement of the abdominal wall with a mesh at the time of stoma formation, has been suggested to decrease the risk of parastomal hernia [2, 3]. Currently, guidelines by the European Hernia Society (EHS) provide a strong recommendation for the use of prophylactic synthetic mesh in the construction of a permanent end colostomy [4]. However, since the development of that recommendation, new randomised trials, as well as long-term follow-up outcomes of existing trials have been published, adding to the existing knowledge [5–7].

In this context, the aim of this study is to investigate the effectiveness of a prophylactic mesh for the construction of a permanent end colostomy. This systematic review and meta-analysis was sponsored by the European Hernia Society, to inform the development of a rapid guideline and potential update of the previous recommendation on the use of prophylactic mesh for permanent end colostomy.

## Methods

The project protocol is available online [8].

### Search Strategy

We updated a previous systematic review with *de novo* evidence search of PubMed from inception up to 16 June 2022 [4]. OpenGrey was no longer operational by the time of the update search. The search syntaxes, date limits, and summary search results are provided in the online appendix [8].

### Study Selection

Study selection was performed by an *ad hoc* evidence research team (AAT, LR) using the platform Rayyan [9]. Both reviewers were blinded to each other’s judgement, and after unblinding, disagreements were resolved through arbitration by the senior author. We considered randomised controlled trials only, comparing the use of prophylactic mesh versus no mesh in the construction of an end colostomy. Overarching inclusion criteria were adult patients receiving an end colostomy for either benign or malignant pathology, in an elective or emergency setting. Outcomes of interest were decided upon within a fully contextualized approach [10].

### Rating the Importance of Outcomes

The importance of outcomes was rated by panel members using the GRADE scale [10]. The classification of outcomes into each of the three categories (not important, important, critical) was made by the steering group under consideration of panel members’ ratings available online [8]. The final rating was the median of panel members’ ratings since there were no substantial deviations from the median.

We considered the importance of outcomes as follows:

1. Clinical diagnosis of parastomal hernia: critical - 7
2. 30 day or in-hospital complications Clavien-Dindo ≥3: critical - 8
3. Surgery for parastomal hernia: critical - 8
4. Quality of life: critical – 9

Primary outcome was quality of life (QoL), while secondary outcomes included major peri-operative morbidity (Clavien-Dindo grade 3 and 4) measured within 30 days from operation or during hospital stay, parastomal hernia diagnosed clinically or radiologically, and surgery for parastomal hernia. An external advisor provided long-term data of their trial [11]. Another two external advisors indicated that longer-term data of their trials have been collected, but they are not yet available for third-party use.

### Setting Minimal Important Differences

The evidence-to-decision framework was set within a fully contextualised approach [10]. An anonymous web-based survey of panel members was performed to define minimal important differences. The results of the survey are available online [8]. The final rating was the median of panel members’ ratings since no substantial deviations from the median were observed.

Under consideration of panel’s responses, the following minimal important differences were set:

1. Clinical diagnosis of parastomal hernia: 50 per 1,000 patients
2. 30 day or in-hospital complications Clavien-Dindo ≥3: 50 per 1,000 patients
3. Surgery for parastomal hernia: 50 per 1,000 patients
4. Quality of life: 25 out of 100 points, or 0.2/0.5 standard deviations (small/moderate difference)

The outcome quality of life was reported with different scales (EORTC QLQ-C30, Short Form 36, Stoma QoL questionnaire); we therefore calculated standardised mean differences. Although no universal cut-off can be applied [12], we considered the above differences in standard deviation units as important for small/moderate difference, based on expert guidance (INGUIDE McMaster guideline methodologist certification program).

### Data Extraction

Outcome data were extracted blindly and independently by two reviewers (AAT, LR), with discrepancies resolved through discussion, or arbitration by the senior author. The data extraction spreadsheet and detailed risk of bias assessments per outcome or group of outcomes with justifications are available online also for third-party use under the Creative Commons license, after approval by the senior author [8].

We used PlotDigitizer to retrieve data from a study report where bar charts were provided instead of absolute values [13].

### Risk of Bias Assessment

We performed *de novo* risk of bias assessments using RoB-2 [14]. Risk of bias assessments were performed by the first author (AAT) and cross checked by the senior author in detail (SAA). For the purposes of outcome-specific risk of bias assessment, outcomes were grouped as follows: 30 day complications Clavien-Dindo ≥3; parastomal hernia and surgery for parastomal hernia; and quality of life. We considered longest-term follow-up data for all outcomes except perioperative complications, with a minimum follow-up of 12 months. Detailed judgements per outcome can be accessed in the online appendix [8].

### Statistical Analysis

We conducted random and fixed effect(s) meta-analyses to synthesise evidence. All outcomes were dichotomous, except for quality of life. We extracted for each group the number of events and the sample size for dichotomous outcomes, and we estimated the study-specific odds ratios along with the corresponding 95% confidence intervals. We used the method of moments estimator, also known as the DerSimonian & Laird estimator for the between study-variance. A continuity correction was applied to the studies with zero-cell counts. For the continuous outcome, we extracted the mean, the sample size, the standard deviation, and we estimated the study-specific standardised mean differences along with the corresponding 95% confidence interval for each group. For what we could not calculate the standard deviation, we used the maximum standard deviation among studies.

We explored heterogeneity via the I^2^ statistic that describes the percentage of the variability of effect estimates that is due to heterogeneity rather than sampling error. We further explored heterogeneity by computing the Q-statistic and the 95% predictive intervals, that indicate the plausible range of effect size values for a future trial. In most of the analyses, it was not possible to check for small study effect either visually by inspecting the symmetry of the funnel plot or statistically by applying Egger’s test, because of an inadequate number of studies. The fixed effect model was applied for all analyses as a sensitivity analysis. Statistical analyses were performed with the R statistical package version 4.0.3 using the *meta* and *metafor* packages.

### Sensitivity Analyses

We performed sensitivity analyses of studies with a minimum follow-up duration of 5 years and compared the effect estimates with studies with shorter follow-up duration. Furthermore, we performed sensitivity analyses of studies at low risk of bias versus high risk/some concerns., as well as subgroup analyses based on the anatomical position of the mesh (retrorectus/intraperitoneal/anterectus). Results from all sensitivity analyses are available in the online appendix [8].

### Assessment of the Certainty of Evidence

We constructed GRADE evidence profiles of certainty for each pairwise comparison separately and for each outcome using GRADEpro GDT [15]. The certainty of evidence is determined by the risk of bias across studies, incoherence, indirectness, imprecision, publication bias and other parameters [16]. To inform calculations of absolute effect differences, we performed proportion meta-analyses of frequencies of baseline risks/effects provided by the source studies; these are available in the online appendix [8]. One study only provided data to allow time-to-event analyses, therefore time-to-event data meta-analysis could not be performed [17].

## Results

We identified 19 reports of 12 randomised trials [2, 5–7, 11, 17–30]. The PRISMA 2020 flow chart is available in the online appendix [8]. Ten trials reported on elective surgery, 11 trials reported primarily on patients with malignancy as background pathology that necessitated construction of a stoma, and all trials reported on the use of synthetic non-absorbable or partially absorbable mesh. Detailed study characteristics are provided in the data extraction sheet available in the online appendix [8].

### Risk of Bias

Three trials provided data regarding QoL, all of which were deemed to be at high risk of bias due to deviations from intended interventions and missing outcome data since only a small proportion of participants managed to complete the QoL questionnaires, while no proper analyses were performed in order to deal with missingness of the outcomes. Peri-operative major morbidity was reported by two trials, both of which were judged at low risk of bias. Finally, parastomal hernia occurrence and surgery for parastomal hernia were handled as a single outcome in regard to the risk of bias assessment. All 12 trials published data concerning parastomal hernia and surgery for parastomal hernia, 6 of which were deemed to be at low risk of bias, 5 as some concerns, and 1 at high risk of bias. The detailed assessment for each domain of the RoB 2 tool per outcome can be accessed in the online appendix [8] and additional considerations regarding overall risk of bias assessment per outcome are provided in the footnotes of the evidence Table 1.

**TABLE 1:** Evidence summary table.

Author(s): Stavros A. Antoniou												
Question: Prophylactic mesh compared to no prophylactic mesh in patients who undergo construction of a permanent end colostomy												
Setting: healthcare/Europe												
Bibliography:												
Certainty assessment							No of patients		Effect		Certainty	Importance
No of studies	Study design	Risk of bias	Inconsistency	Indirectness	Imprecision	Other considerations	Prophylactic mesh	No prophylactic mesh (%)	Relative (95% CI)	Absolute (95% CI)		
**Major morbidity (30 day) (assessed with: Clavien-Dindo ≥3)**												
2^a^	Randomised trials	Not serious	Not serious	Not serious	Very serious^b^	None	30/135 (22.2%)	7.3	**OR 0.77** (0.45–1.30)	**16 fewer per 1,000** (from 39 fewer to 20 more)	⊕⊕○○ Low	CRITICAL
								23.8		**44 fewer per 1,000** (from 115 fewer to 51 more)		
								55.3		**65 fewer per 1,000** (from 195 fewer to 64 more)		
**Parastomal hernia (follow-up: mean 5 years; assessed with: physical examination)**												
12	Randomised trials	Not serious^c^	Serious^d^	Not serious^e^	Not serious	None^f^	123/457 (26.9%)	36.3%	**OR 0.33** (0.18–0.62)	**205 fewer per 1,000** (from 270 fewer to 102 fewer)	⊕⊕⊕○ Moderate	CRITICAL
								45.3%		**238 fewer per 1,000** (from 323 fewer to 114 fewer)		
								54.7%		**262 fewer per 1,000** (from 368 fewer to 119 fewer)		
**Surgery for parastomal hernia (follow-up: mean 5 years)**												
3	Randomised trials	Serious^g^	Not serious	Not serious^h^	Serious^i^	None	3/115 (2.6%)	2.5%	**OR 0.18** (0.06–0.59)	**20 fewer per 1,000** (from 23 fewer to 10 fewer)	⊕⊕○○ Low	CRITICAL
								5.0%		**41 fewer per 1,000** (from 47 fewer to 20 fewer)		
								9.5%		**76 fewer per 1,000** (from 89 fewer to 37 fewer)		
**Quality of life (follow-up: range 1 year–5 years; assessed with: EORTC QLQ-C30, Short Form 36, Stoma QoL questionnaire)**												
3	Randomised trials	Very serious^j^	Not serious	Not serious	Not serious	None	266	267	-	SMD **0.03 SD higher** (0.14 lower to 0.2 higher)	⊕⊕○○ Low	CRITICAL

**CI**, confidence interval; **OR**, odds ratio; **SMD**, standardised mean difference.

**Explanations**.

^a^ The top row in each set of absolute effect estimates represents estimated difference in low baseline risk patients, the middle row represents estimated difference in moderate baseline risk patients, and the bottom row represents estimated difference in high baseline risk patients.

^b^ Very wide confidence interval crossing lower and upper decision thresholds, unless low baseline risk of major morbidity.

^c^ Several studies with some concerns. Sensitivity (random effects) and meta-regression analysis did not indicate substantially different effect estimates of studies at low risk of bias versus those with some concerns (see sensitivity analyses in online appendix). Therefore, we did not downgrade the certainty of evidence in this domain.

^d^ Substantial heterogeneity (I^2^ = 73%), however we did not downgrade for both heterogeneity and imprecision, because the former is mitigated by the random effects model and is addressed by the domain of imprecision.

^e^ Sensitivity (random effects) and meta-regression analysis did not indicate substantial difference between studies with follow-up ≥5 years versus <5 years (panel-set threshold for minimal clinical importance). We therefore considered the pooled comparative outcome irrespective of duration of follow-up.

^f^ Asymmetrical funnel plot and significant evidence of publication bias on Egger’s test (*p* = 0.0002) in summary analysis; however, we did not double-downgrade for both heterogeneity and publication bias, because of overlapping effects.

^g^ Several studies with some concerns. Sensitivity (random effects) and meta-regression analysis did not indicate substantially different effect estimates of studies at low risk of bias versus those with some concerns (see sensitivity analyses in online appendix). However, visual inspection of sensitivity analyses suggest that there may be inflated effect estimates in studies with some concerns/high risk, that is statistically undetectable. Therefore, we downgraded the certainty of evidence in this domain by one level.

^h^ Sensitivity (random effects) and meta-regression analysis indicated substantial difference between studies with follow-up ≥5 years versus <5 years. We therefore considered studies reporting ≥5 year data only (panel-set threshold for minimal clinical importance).

^i^ Downgraded due to few events, and because the confidence interval is crossing lower decision threshold when highest baseline risk is considered.

^j^ Mostly due to missing data.

### Quality of Life

**FIGURE 1:**
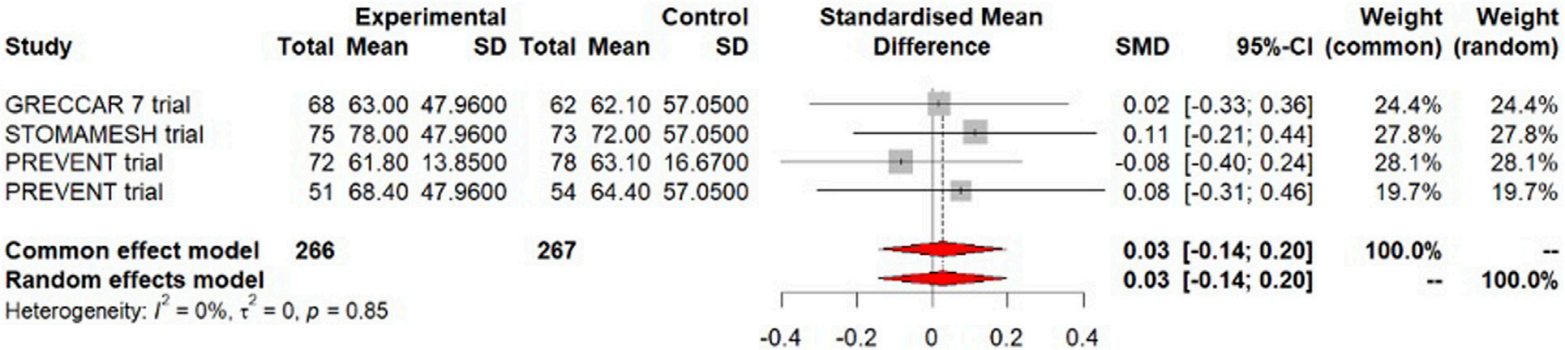
Forest plot of meta-analysis for the outcome quality of life.

QoL was our primary outcome and was reported by 3 trials with a total of 533 participants having completed the QoL questionnaires (GRECCAR-7, STOMAMESH, and PREVENT trials) [5, 18, 27]. No clinically relevant difference was observed between patients with a prophylactic mesh and patients without mesh in the overall QoL scores (SMD = 0.03, 95% CI [−0.14 to 0.2], low certainty of evidence). There was no evidence of statistical heterogeneity (I^2^ = 0%; Figure 1).

### Parastomal Hernia

**FIGURE 2:**
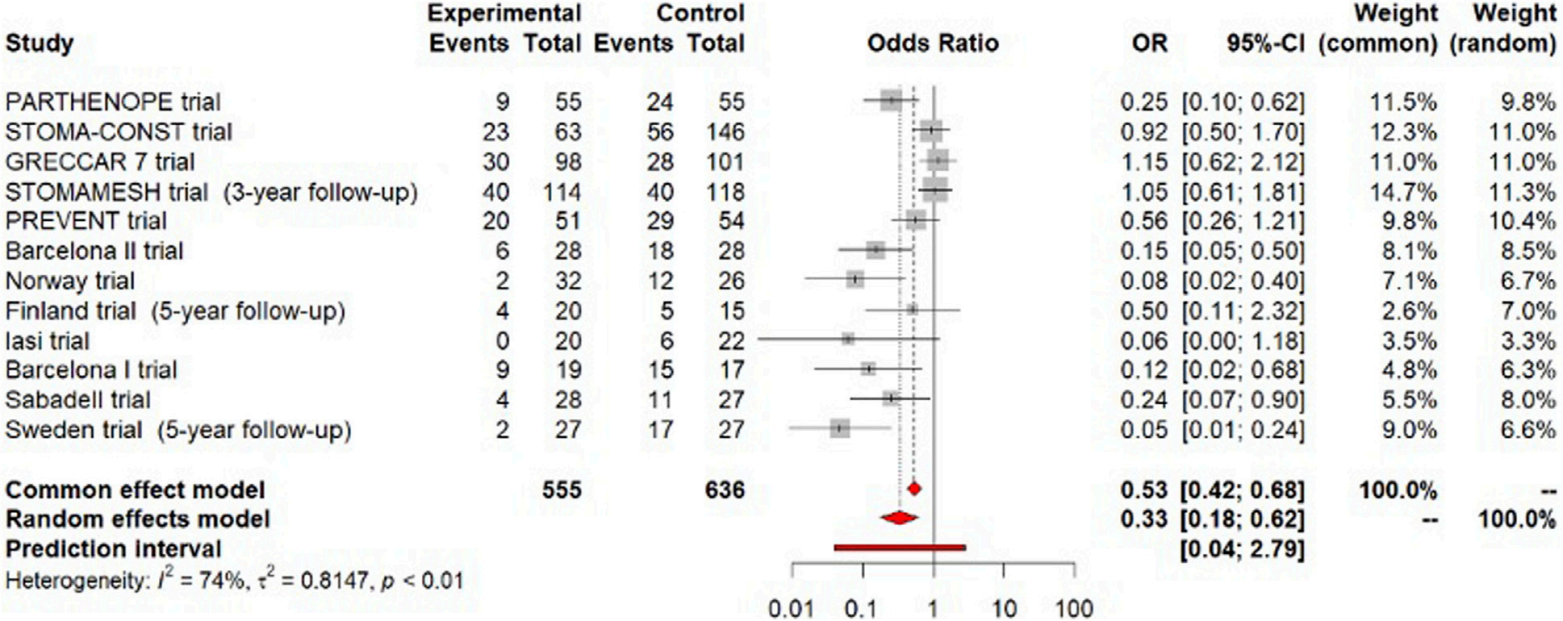
Forest plot of meta-analysis for the outcome parastomal hernia.

All 12 included trials reported on parastomal hernia incidence. Meta-analysis of those trials, including data from 1.191 patients, showed a clinically significant reduction of the incidence of parastomal hernia in the group of patients offered a prophylactic mesh for stoma construction (OR = 0.33, 95% CI [0.18–0.62], moderate certainty of evidence), with considerable evidence of statistical heterogeneity (I^2^ = 74%; Figure 2). This corresponds to an absolute difference of 102–270 fewer patients per 1,000 (low risk group), 114–323 fewer patients per 1,000 (moderate risk group), and 119–368 fewer patients per 1,000 (high risk group).

Meta-analysis of 9 trials reporting the incidence of parastomal hernia within a follow-up period ≤5 years, demonstrated a clinically significant reduction in favour of the mesh arm (OR = 0.35, 95% CI [0.17–0.72]). Evidence of statistical heterogeneity was considerable (I^2^ = 75%).

When examining the incidence of parastomal hernia in the long-term, by synthesising data from 3 trials that reported on long-term follow-up (≥5 years) of 194 patients [6, 11, 21], we found a clinically relevant risk reduction with the use of a prophylactic mesh (OR = 0.26, 95% CI [0.06–1.16]), although with considerable evidence of statistical heterogeneity (I^2^ = 73%). Sensitivity (random effects) and meta-regression analysis did not indicate substantial difference between studies with follow-up ≥5 years versus <5 years. Therefore, we considered the pooled comparative outcome irrespective of duration of follow-up.

### Major Peri-Operative Morbidity

**FIGURE 3:**
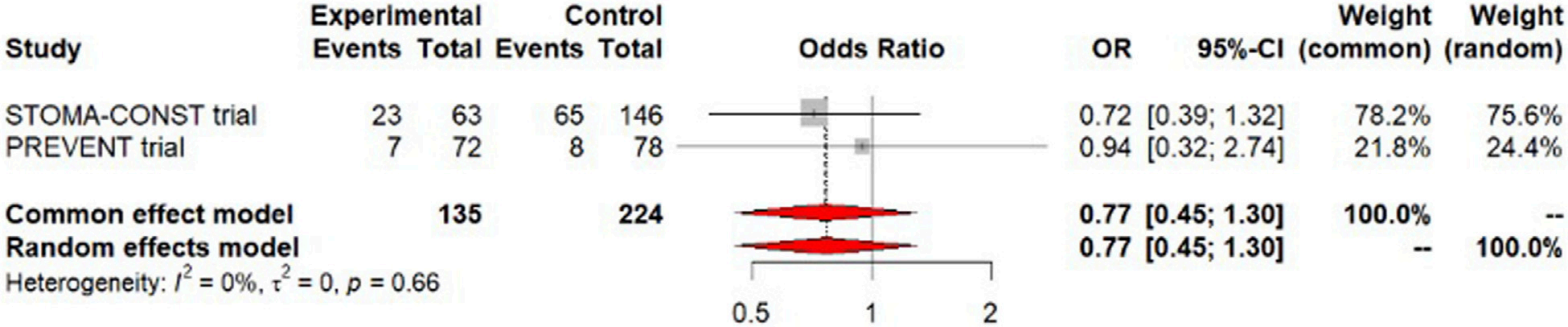
Forest plot of meta-analysis for the outcome major morbidity.

Meta-analysis of 2 trials (STOMA-CONST and PREVENT trials) with a total number of 359 patients found no significant difference in Clavien-Dindo grade 3 and 4 adverse events after surgery with or without the addition of a prophylactic mesh (OR = 0.77, 95% CI [0.45–1.30], low certainty of evidence). There was negligible evidence of statistical heterogeneity (I^2^ = 0%; Figure 3). This corresponds to an absolute difference of 39 fewer to 20 more patients per 1,000 (low risk group), 115 fewer to 51 more patients per 1,000 (moderate risk group), and 195 fewer to 64 more patients per 1,000 (high risk group).

### Surgery for Parastomal Hernia

**FIGURE 4:**
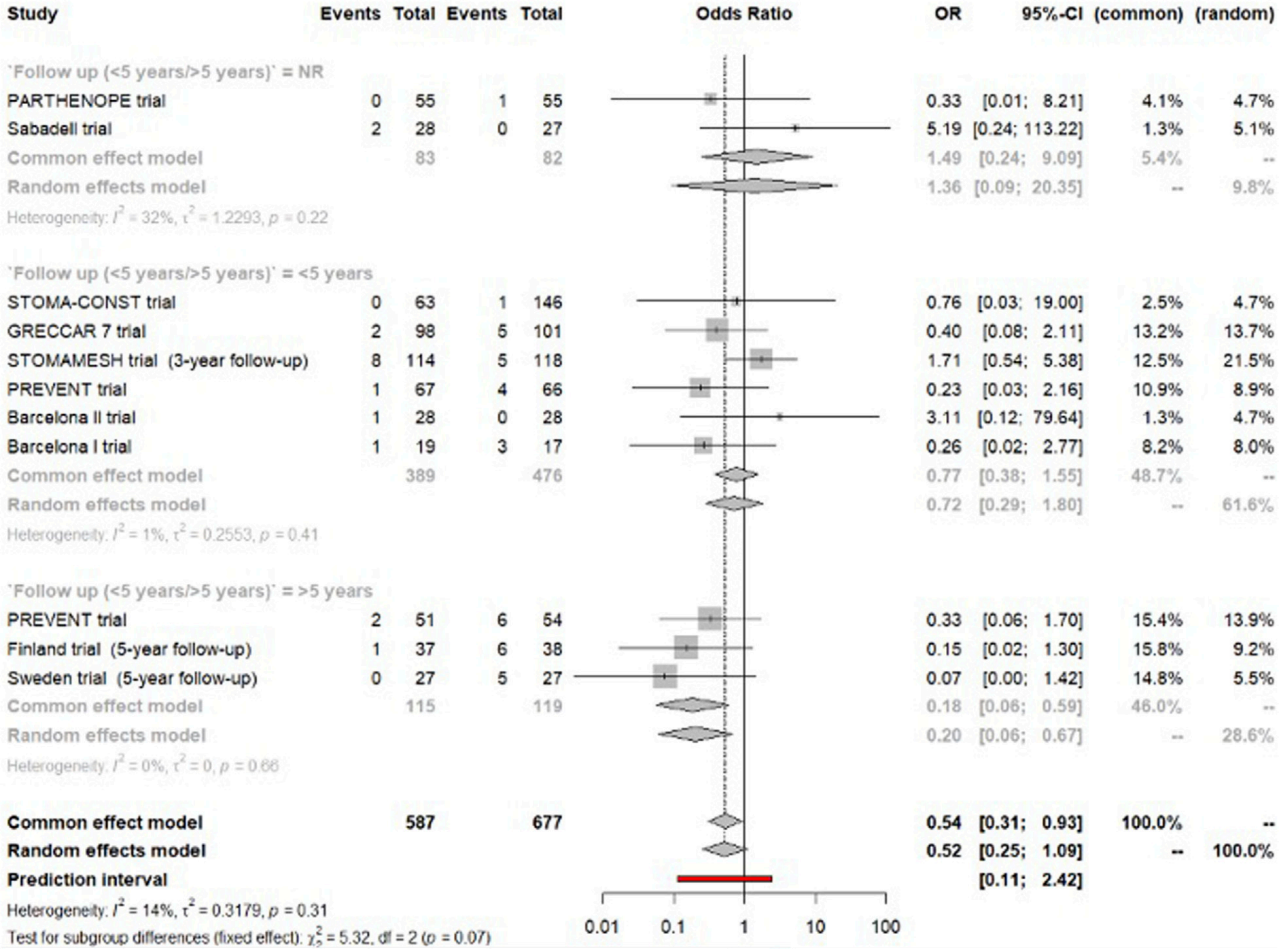
Forest plot of meta-analysis for the outcome surgery for parastomal hernia.

Meta-analysis of 10 trials found a non-clinically significant reduction in the risk for parastomal hernia surgical intervention in the mesh group (OR = 0.52, 95% CI [0.25–1.09]). Evidence of statistical heterogeneity was low (I^2^ = 14%, Figure 4).

Six trials reported on parastomal hernia operation within a follow-up period <5 years [5, 7, 18, 22, 23, 28]. Quantitative synthesis showed no difference between the two arms (OR = 0.72, 95% CI [0.29–1.80]). The statistical heterogeneity was negligible (I^2^ = 1%).

Sensitivity (random effects) and meta-regression analysis indicated substantial difference between studies with follow-up ≥5 years versus <5 years. We therefore considered studies reporting ≥5 year data only (panel-set threshold for minimal clinical importance).

Meta-analysis of long-term follow-up data by 3 trials [6, 11, 21], reporting on a total of 234 patients that were followed for a mean period of 5 years, demonstrated a non-clinically significant reduction in parastomal hernia surgery for patients in the mesh group (OR = 0.20, 95% CI [0.06–0.59], low certainty of evidence). No statistical heterogeneity was observed (I^2^ = 0%). This corresponds to an absolute difference of 10–23 fewer patients per 1,000 (low risk group), 20–47 fewer patients per 1,000 (moderate risk group), and 37–89 fewer patients per 1,000 (high risk group).

Detailed statistical analyses are provided in the online appendix [8].

## Discussion

### Summary of Main Findings

Meta-analysis of RCTs data showed no difference in QoL between prophylactic mesh and no mesh for primary stoma construction. With regard to parastomal hernia, the use of prophylactic synthetic mesh resulted in a significant risk reduction of the incidence of the event, according to data from all available RCTs, irrespective of the follow-up period (primary analysis). The same result was encountered after sensitivity analysis of short-term follow-up data (<5 years). Meta-analysis of RCTs reporting on long-term follow-up data (≥5 years) did not indicate significant statistical deviation from the primary analysis.

The effect sizes and the relative effect estimates were not consistent across trials, with 3 (STOMA-CONST, GRECCAR 7 and STOMAMESH) finding no effect. Sensitivity analyses that accounted for risk of bias, method of diagnosis, duration of follow-up and mesh position could not explain this discrepancy of estimated effects. This suggests that other understudied parameters are implicated. These may include technical details of the operation, the position of the stoma, the size and construction characteristics of the mesh, and perioperative management.

Regarding surgery for parastomal hernia, sensitivity analyses showed no difference in the risk for parastomal hernia operation between the two arms in the short-term (<5 years follow-up), however in the long-term (≥5 years follow-up), meta-analysis showed that patients in the mesh arm had a significantly reduced risk for parastomal hernia operation. Major peri-operative morbidity was similar between the two arms.

### Certainty of the Evidence

Only a small number of RCTs that have been conducted and included in our systematic review have thoroughly investigated comparative QoL scores between patients offered a prophylactic synthetic mesh and patients with no mesh placement at the time of an end colostomy formation. Our analysis and appraisal of the evidence demonstrated that prophylactic mesh may result in little to no difference in QoL (SMD 0.03 SD higher [0.14 lower to 0.2 higher]). The certainty of the evidence was low due to very serious risk of bias, which resulted from deviations from intended interventions and missing outcome data.

Regarding parastomal hernia, even though primary analysis and sensitivity analysis of short-term follow-up data yielded both a statistically significant and clinically relevant result in favour of the mesh group, this was not statistically demonstrated in the long-term sensitivity analysis. As a result, one could argue that prophylactic mesh might not actually prevent parastomal hernia, but rather delays its onset. However, given the small sample size available for the sensitivity analysis (359 patients), there is high probability for type II error. Nonetheless, the results of the long-term sensitivity analysis seem to align with those of the primary analysis, since the CIs of both analyses are overlapping. As a result, we assumed that the effect estimates of the primary analysis apply to the sensitivity analysis of the long-term data, hence we did not downgrade the certainty of the evidence for imprecision. We therefore hypothesize that the effect of the prophylactic mesh is likely maintained in the long-term.

The same principle was applied for the sensitivity analyses of the outcome “surgery for parastomal hernia.” Primary analyses showed no benefit of prophylactic mesh over no mesh for the risk reduction of parastomal hernia surgery. Sensitivity analysis of long-term follow-up data demonstrated a clinically significant risk reduction in favour of the mesh group, only in small and moderate baseline risk patients. With high baseline risk included, the CI is crossing the minimally importance difference threshold. For this reason, and due to small sample size available for the sensitivity analysis, we downgraded our judgement on the certainty of the evidence for imprecision [8]. Subsequently, we assume that prophylactic synthetic mesh placement during construction of a permanent end colostomy will likely result in little or no difference in parastomal hernia surgery.

The less well-studied outcome of this systematic review was major peri-operative morbidity, Clavien-Dindo classification ≥3. Data extraction was challenging since most of the included RCTs did not properly report peri-operative adverse events or whether some of them were managed conservatively or not. Consequently, only data from trials that thoroughly recorded peri-operative adverse events and their management were utilised in our analysis, which demonstrated little or no difference in major morbidity between patients assigned to mesh or no mesh arm. The certainty of the evidence was low due to imprecision since our analysis yielded a very wide confidence interval that crossed both lower and upper decision thresholds [8].

Summary appraisal of the certainty of the evidence is provided in Table 1. Table 2 provides informative statements on relative effectiveness.

**TABLE 2:** Summary of findings table.

Prophylactic mesh compared to no prophylactic mesh in patients who undergo construction of a permanent end colostomy						
Patient or population: patients who undergo construction of a permanent end colostomy						
Setting: healthcare/Europe						
Intervention: prophylactic mesh						
Comparison: no prophylactic mesh						
Outcomes	Anticipated absolute effects^*^ (95% CI)		Relative effect (95% CI)	№ of participants (studies)	Certainty of the evidence (GRADE)	Comments
	Risk with no prophylactic mesh	Risk with prophylactic mesh				
Major morbidity (30 day) (Major morbidity) assessed with: Clavien-Dindo ≥3	**Low**		**OR 0.77** (0.45–1.30)	359 (2 RCTs)^a^	⊕⊕○○ Low^b^	Prophylactic mesh may result in little to no difference in the risk of major morbidity in the elective setting
	73 per 1,000	**57 per 1,000** (34–93)				
	**Moderate**					
	238 per 1,000	**194 per 1,000** (123–289)				
	**High**					
	553 per 1,000	**488 per 1,000** (358–617)					
Parastomal hernia (PSH) assessed with: physical examination follow-up: mean 5 years	**Low**		**OR 0.33** (0.18–0.62)	997 (12 RCTs)	⊕⊕⊕○ Moderate^c^ ^,^ ^d^ ^,^ ^e^ ^,^ ^f^	Prophylactic mesh likely results in a reduction in parastomal hernia
	363 per 1,000	**158 per 1,000** (93–261)				
	**Moderate**					
	453 per 1,000	**215 per 1,000** (130–339)				
	**High**					
	547 per 1,000	**285 per 1,000** (179–428)					
Surgery for parastomal hernia (Surgery for PSH) follow-up: mean 5 years	**Low**		**OR 0.18** (0.06–0.59)	234 (3 RCTs)	⊕⊕○○ Low^g^ ^,^ ^h^ ^,^ ^i^	Prophylactic mesh may result in little to no difference in surgery for parastomal hernia
	25 per 1,000	**5 per 1,000** (2–15)				
	**Moderate**					
	50 per 1,000	**9 per 1,000** (3–30)				
	**High**					
	95 per 1,000	**19 per 1,000** (6–58)					
Quality of life (QoL) assessed with: EORTC QLQ-C30, Short Form 36, Stoma QoL questionnaire follow-up: range 1 year–5 years	-	SMD **0.03 SD higher** (0.14 lower to 0.2 higher)	-	533 (3 RCTs)	⊕⊕○○ Low^j^	Prophylactic mesh may result in little to no difference in quality of life

^*^ The risk in the intervention group (and its 95% confidence interval) is based on the assumed risk in the comparison group and the relative effect of the intervention (and its 95% CI).

**CI**, confidence interval; **OR**, odds ratio; **SMD**, standardised mean difference.

**GRADE Working Group grades of evidence**.

**High certainty:** we are very confident that the true effect lies close to that of the estimate of the effect.

**Moderate certainty:** we are moderately confident in the effect estimate: the true effect is likely to be close to the estimate of the effect, but there is a possibility that it is substantially different.

**Low certainty:** our confidence in the effect estimate is limited: the true effect may be substantially different from the estimate of the effect.**Very low certainty:** we have very little confidence in the effect estimate: the true effect is likely to be substantially different from the estimate of effect.

**Explanations**.

^a^ The top row in each set of absolute effect estimates represents estimated difference in low baseline risk patients, the middle row represents estimated difference in moderate baseline risk patients, and the bottom row represents estimated difference in high baseline risk patients.

^b^ Very wide confidence interval crossing lower and upper decision thresholds, unless low baseline risk of major morbidity.

^c^ Several studies with some concerns. Sensitivity (random effects) and meta-regression analysis did not indicate substantially different effect estimates of studies at low risk of bias versus those with some concerns (see sensitivity analyses in online appendix). Therefore, we did not downgrade the certainty of evidence in this domain

^d^ Substantial heterogeneity (I^2^ = 73%), however we did not downgrade for both heterogeneity and imprecision, because the former is mitigated by the random effects model and is addressed by the domain of imprecision.

^e^ Sensitivity (random effects) and meta-regression analysis did not indicate substantial difference between studies with follow-up ≥5 years versus <5 years (panel-set threshold for minimal clinical importance). We therefore considered the pooled comparative outcome irrespective of duration of follow-up.

^f^ Asymmetrical funnel plot and significant evidence of publication bias on Egger’s test (*p* = 0.0002) in summary analysis; however, we did not double- downgrade for both heterogeneity and publication bias, because of overlapping effects.

^g^ Several studies with some concerns. Sensitivity (random effects) and meta-regression analysis did not indicate substantially different effect estimates of studies at low risk of bias versus those with some concerns (see sensitivity analyses in online appendix). However, visual inspection of sensitivity analyses suggest that there may be inflated effect estimates in studies with some concerns/high risk, that is statistically undetectable. Therefore, we downgraded the certainty of evidence in this domain by one level.

^h^ Sensitivity (random effects) and meta-regression analysis indicated substantial difference between studies with follow-up ≥5 years versus <5 years. We therefore considered studies reporting ≥5 year data only (panel-set threshold for minimal clinical importance).

^i^ Downgraded due to few events, and because the confidence interval is crossing lower decision threshold when highest baseline risk is considered.

^j^ Mostly due to missing data.

### Limitations

Our study was subject to certain limitations, the most important of which was the heterogeneity observed between the included trials. Even though we attempted to investigate the sources of heterogeneity conducting sensitivity analyses according to the duration of follow-up (short-term/long-term), risk of bias, anatomical position of the mesh, there might be yet some extent of residual heterogeneity arising from the setting of primary stoma construction (emergency/elective/mixed), the experience of the participating institutions, the level of expertise of the surgeons, and the criteria applied for clinical diagnosis of parastomal hernia. Furthermore, there were not enough data -neither by the included RCTs nor by observational studies searched within the literature-to construct a better informed analysis of the major peri-operative complications expected in the short-term or the long-term by the use of prophylactic synthetic meshes. Finally, hazard ratios for outcomes like parastomal hernia and parastomal hernia surgery were not provided and as such a time-to-event meta-analysis could not be performed in order to investigate possible effects of prophylactic mesh over time.

### Conclusion

In conclusion, prophylactic synthetic mesh placement at the time of end colostomy construction is likely associated with a reduced risk for parastomal hernia and may confer similar risk of peri-operative major morbidity compared to no mesh placement. There may be no difference in quality of life and surgical repair of parastomal hernia with the use of either approach.

## Data Availability

The datasets presented in this study can be found in online repositories. The names of the repository/repositories and accession number(s) can be found below: https://osf.io/k4sh8/?view_only&equals;b7e9c926976640d288878d1a7c2ee38f.
